# Pediatric anesthesia practices during the COVID‐19 pandemic: A retrospective cohort study

**DOI:** 10.1002/hsr2.979

**Published:** 2022-12-11

**Authors:** Jordan M. Ruby, Alex Illescas, Haoyan Zhong, Kathryn R. DelPizzo, Jashvant Poeran, Jiabin Liu, Crispiana Cozowicz, Stavros G. Memtsoudis

**Affiliations:** ^1^ Department of Anesthesiology, Critical Care & Pain Management Hospital for Special Surgery New York New York USA; ^2^ Department of Anesthesiology Weill Cornell Medicine New York New York USA; ^3^ Department of Population Health Science & Policy/Orthopedics, Icahn School of Medicine at Mount Sinai Institute for Healthcare Delivery Science New York New York USA; ^4^ Department of Anesthesiology, Perioperative Medicine and Intensive Care Medicine Paracelsus Medical University Salzburg Austria; ^5^ Department of Health Policy and Research Weill Cornell Medical College New York New York USA

**Keywords:** anesthesia, COVID‐19, fracture, pediatric

## Abstract

**Background and Aims:**

The onset of the coronavirus 2019 (COVID‐19) pandemic brought together the American Society of Regional Anesthesia and Pain Medicine (ASRA) and the European Society of Regional Anaesthesia and Pain Therapy (ESRA) to release a joint statement on anesthesia use. Their statement included a recommendation to use regional anesthesia whenever possible to mitigate the risk associated with aerosolizing procedures. We sought to examine the utilization of anesthesia in pediatric patients undergoing a surgical procedure for fractures or ligament repairs before and during COVID‐19.

**Methods:**

Using the Premier Health Database, we retrospectively analyzed pediatric patients undergoing a surgical intervention for fractures or ligament repair before and during COVID‐19. We sought to determine if there were differences in anesthesia use among this cohort during the two time periods. Fracture groups included shoulder and clavicle, humerus and elbow, forearm and wrist, hand and finger, pelvis and hip, femur and knee, leg and ankles, and foot and toes. Ligament procedures included surgical intervention for the anterior cruciate ligament and ulnar collateral ligament repair.

**Results:**

We identified a total of 5935 patients undergoing a surgical procedure for fractures or ligament repairs before and during COVID‐19. After exclusion for unknown anesthesia use, 2,807 patients were included in our cohort with 81.5% (*n* = 2288) of patients undergoing a procedure under general anesthesia, 6.4% (*n* = 181) under regional anesthesia, and 12.0% (*n* = 338) under combined general‐regional anesthesia. There did not appear to be a significant difference in the type of anesthesia used before and during COVID‐19 (*p* = 0.052).

**Conclusions:**

Our study did not identify a difference in anesthesia use before and during COVID‐19 among pediatric patients undergoing a surgical procedure. Further studies should estimate the change in anesthesia used during the time period when elective procedures were resumed.

## 1 INTRODUCTION

The clinical practice changed dramatically with the onset of the coronavirus 2019 (COVID‐19) pandemic with elective surgical cases being postponed and with medical societies offering guidance for practice changes.^1^, ^2^, ^3^ The American Society of Regional Anesthesia and Pain Medicine (ASRA) and the European Society of Regional Anaesthesia and Pain Therapy (ESRA) published a joint statement in March 2020 recommending the utilization of regional anesthesia whenever possible to negate the risk that is undertaken when aerosolizing procedures, like endotracheal intubations, are performed.^4^ When the United States emerged from the initial surge of the pandemic, elective surgery resumed under significantly different circumstances.^5^ The virus remained an omnipresent part of daily practice necessitating rigorous personal protective equipment precautions and strict testing measures undertaken before surgery.^6^, ^7^ While studies attempted to detail the impact on actual anesthetic practice, data, especially in the pediatric population, remains scarce.^8^, ^9^

To date, there has been no population‐based inquiry with regard to the utilization of regional anesthesia in lieu of general anesthesia in children during the COVID‐19 pandemic. We, therefore, hypothesized that there would be an increase in the application of regional anesthesia in pediatric patients temporal to the COVID‐19 pandemic in comparison to the year prior given the aforementioned societal recommendations.

## 2 METHODS

In this retrospective cohort study, we analyzed pediatric patients (<21 years old) within the Premier Healthcare Database who underwent a surgical intervention for fractures or ligament repairs such as anterior cruciate ligament (ACL) or ulnar collateral ligament (UCL) during March to June of 2019 (pre‐COVID‐19) and 2020 (during the initial wave of COVID‐19 in the United States). The Premier Healthcare database is an all‐payer database containing information for approximately 20%–25% of inpatient discharges in the United States from over 700 participating hospitals. The database contains detailed information on patient characteristics as well as hospital characteristics such as comprehensive billing, cost, device, medication, and procedure information.

Patient records with an International Classification of Diseases, 10th Revision, Clinical Modification (ICD‐10‐CM) procedure code or Current Procedural Terminology (CPT) code for surgical intervention of a fracture or ACL/UCL repair were included in this cohort (Appendix: Table A1). Procedures included surgical intervention for fractures separated by the location of the fracture (shoulder and clavicle, humerus and elbow, forearm and wrist, hand and finger, pelvis and hip, femur and knee, leg and ankle, and foot and toe) and surgical intervention for ACL/UCL repair.^10^, ^11^ Using billing data, we identified the anesthesia type for each patient. Those without billing information for anesthesia type were excluded from the analysis (*n* = 3128).

Our primary study goal was to determine if there were differences in anesthesia use for pediatric fractures and ACL/UCL repair during the COVID‐19 pandemic in comparison to the prior year. We compared general, regional, and combined general‐regional use before and during COVID‐19. We compared the same monthly time frame (March to June) for both 2019 and 2020.^12^ Other patient and healthcare characteristics were assessed including age, sex, race, admission type, insurance type, Charlson–Deyo comorbidity index,^13^ region, hospital location, bed size, teaching status, and location of the fracture or ACL/UCL repair.

A *χ* ^2^ test was used to assess differences in anesthesia use before and during COVID‐19. For the remaining variables, *χ* ^2^ and Mann–Whitney *U*‐tests were used to assess 2019–2020 differences in terms of categorical (reported by number and percentage) and continuous (reported by median and interquartile range) variables, respectively. All statistical analyses were conducted using SAS version 9.4 (SAS Institute). A *p*‐value of <0.05 was determined as statistically significant.

## 3 RESULTS

**Figure 1:**
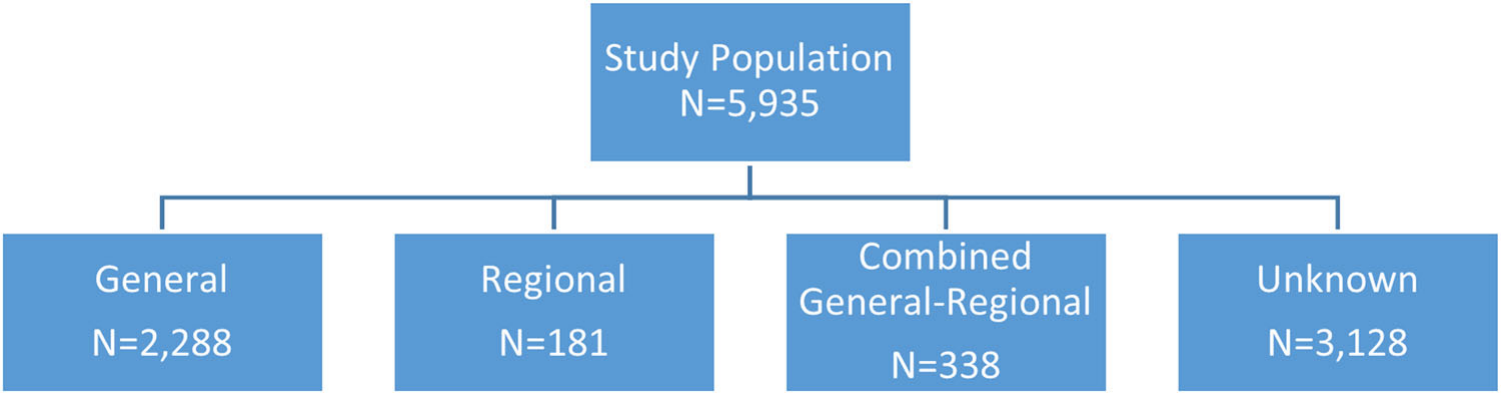
Flowchart used to identify the study population and anesthesia subgroupings

We identified a total of 5935 patients under 21 years old who underwent a surgical intervention for fracture or ACL/UCL repair during 2019 (pre‐COVID‐19) and 2020 (during COVID‐19). Of those, 3128 did not have information on anesthesia type and were excluded (Figure 1). Our final study population consisted of 2807 patients, of which 81.5% (*n* = 2288) of procedures were performed under general anesthesia, 6.4% (*n* = 181) were performed under regional anesthesia, and 12.0% (*n* = 338) were performed under combined general‐regional anesthesia. We identified an uptick in regional anesthesia use (5.6%–7.7%) and a decline in combined anesthesia use (12.7%–11.2%) before and during COVID‐19; however, we did not identify a significant difference in terms of the type of anesthesia used (Table 1; *p* = 0.05).

**Table 1:** Patient and hospital level characteristics in the Premier database before and during COVID‐19

	Pre‐COVID‐19		During COVID‐19		*p* Value
	*N* = 1604	(%)	*N* = 1203	(%)	
Anesthesia type					0.05
General	1312	81.8	976	81.1	
Regional	89	5.6	92	7.7	
Combined	203	12.7	135	11.2	
Age, median (IQR)	16 (13–18)		16 (13–19)		0.28
Sex					0.32
Female	663	41.3	475	39.5	
Male	941	58.7	728	60.5	
Race					0.27
Black	214	13.3	136	11.3	
White	946	59.0	729	60.6	
Other	444	27.7	338	28.1	
Admission type					0.003
Emergency	368	22.9	325	27.0	
Urgent	85	5.3	68	5.7	
Elective	886	55.2	581	48.3	
Trauma center	171	10.7	164	13.6	
Unknown/other	94	5.9	65	5.4	
Insurance					0.87
Commercial	783	48.8	584	48.6	
Medicaid	619	38.6	455	37.8	
Medicare	4	0.3	4	0.3	
Uninsured	77	4.8	68	5.7	
Unknown	121	7.5	92	7.7	
Comorbidity index					0.95
0	1436	89.5	1073	89.2	
1	156	9.7	120	10.0	
2+	12	0.8	10	0.8	
Region					0.67
Midwest	424	26.4	304	25.3	
Northeast	153	9.5	107	8.9	
South	809	50.4	635	52.8	
West	218	13.6	157	13.1	
Hospital location					0.46
Rural	233	14.5	163	13.6	
Urban	1371	85.5	1040	86.5	
Hospital bed size					0.57
0–299	452	28.2	353	29.3	
300–499	460	28.7	355	29.5	
500+	692	43.1	495	41.2	
Hospital teaching status					0.21
Nonteaching	583	36.4	465	38.7	
Teaching	1021	63.7	738	61.4	

Abbreviations: COVID‐19, coronavirus 2019; IQR, interquartile range.

Overall, there was nearly a 25% decrease in surgical intervention procedures during the COVID‐19 study period compared to the year prior. Elective procedures decreased during the COVID‐19 study period (55.2% in 2019, 48.3% in 2020) while emergency admissions (22.9% in 2019, 27.0% in 2020) and trauma center admissions (10.7% in 2019, 13.6% in 2020) increased (*p* = 0.003). All other variables including age, sex, and race did not significantly differ between the study periods. The most common types of fractures involved the leg and ankle (Table 2; 44.8% in 2019, 49.4% in 2020; *p* = 0.02) and femur and knee (24.3% in 2019, 23.8% in 2020; *p* ≤ 0.0001).

**Table 2:** Procedures performed before and during COVID‐19

	Pre‐COVID‐19		During COVID‐19		*p* Value
	*N*	(%)	*N*	(%)	
Shoulder and clavicle	71	4.4	52	4.3	0.89
Humerus and elbow	87	5.4	80	6.7	0.17
Forearm and wrist	106	6.6	94	7.8	0.22
Hand and finger	72	4.5	63	5.2	0.36
Pelvis and hip	107	6.7	73	6.1	0.52
Femur and knee	389	24.3	286	23.8	0.77
Leg and ankle	718	44.8	594	49.4	0.02
Foot and toe	76	4.7	52	4.3	0.60
ACL/UCL	160	10.0	70	5.8	<0.0001

Abbreviations: ACL, anterior cruciate ligament; COVID‐19, coronavirus 2019; UCL, ulnar collateral ligament.

## 4 DISCUSSION

In this population‐based study, we examined whether there was a change in the anesthetic practice patterns in the Premier database for US pediatric patients during the COVID‐19 pandemic in accordance with recommendations from ASRA and ESRA. Our data set demonstrates no significant difference in practice despite these recommendations. It is also interesting to note that there was no variation in practice despite the historical precedent of endotracheal intubation being an independent risk factor for nosocomial infections during the 2002 outbreak of severe acute respiratory syndrome.^14^

Previous large database analyses have demonstrated the safety of regional anesthesia in pediatric patients. However, these analyses also demonstrate that the vast majority of neuraxial and peripheral nerve blocks are performed while the child is under general anesthesia with presumably an advanced airway (endotracheal tube or laryngeal mask airway). Additionally, most neuraxial blocks in these analyses were caudal blocks, which have limited application in older children and adolescents.^15^, ^16^, ^17^ Furthermore, practitioners may be unfamiliar with using an epidural or subarachnoid block as a primary anesthetic in children. It should be noted, however, that there is a growing use of spinal anesthesia in lieu of general anesthesia for infants in the appropriate setting.^18^, ^19^

An additional database study reaffirmed the safety of upper extremity nerve blocks in children. However, this analysis also suggested that the application of such an anesthetic is not widely implemented by the pediatric anesthesia community in the United States, as one participating center accounted for nearly half of all the upper extremity nerve blocks in the data set.^20^ Lack of training and experience in advanced upper extremity regional anesthesia techniques as well as a limitation in available resources (i.e., ultrasound machine) could explain why a near majority of upper extremity blocks were performed by this single institution.^21^, ^22^, ^23^

The challenge of implementing best practices in the case of regional anesthesia has been well documented, particularly in the case of adult patients.^24^, ^25^, ^26^ This could certainly extrapolate to the pediatric anesthesia community as a limitation in overhauling institutional practice norms during the COVID‐19 pandemic as most pediatric regional blocks do not serve as a primary anesthetic.^15^, ^16^, ^17^ Taking all the aforementioned factors into consideration, it is perhaps not surprising that there was no change in anesthetic practice patterns for pediatric patients in the United States during the initial months of the pandemic when caution was of the utmost importance and reliable information was often times difficult to ascertain.

There are a number of limitations to our study. Foremost, this is a population‐based analysis from the Premier database, which may not be representative of the population as a whole. Additionally, there was overall less elective surgery during the initial months of the pandemic. This could have affected our results as elective surgeries that are amendable to a regional anesthetic, such as ACL repair, were less common in the 2020 data set. Practice patterns during this time may have also been affected by the regional prevalence and local attitudes toward COVID‐19. Finally, although the ASRA and ESRA joint statement was likely made regarding adult patients, the recommendation should be equally applicable to pediatric patients. Further studies could examine whether there were practice changes after the initial surge of the pandemic with the resumption of elective surgery. Trends for the use of regional anesthesia in pediatric patients can also be examined during subsequent COVID‐19 surges (delta and omicron variants) during which time elective surgery could continue depending on state and municipal regulations.

## 5 CONCLUSION

In summary, our study demonstrates no significant difference in the utilization of regional anesthesia in pediatric patients when comparing prepandemic with pandemic anesthetic practice patterns. The significance of these findings highlights how societal recommendations and historical precedent may not influence pediatric anesthesia practice models. Further studies could evaluate if there was any change in anesthetic techniques with the widespread resumption of elective surgery during the new normal of the COVID‐19 pandemic.

## AUTHOR CONTRIBUTIONS

**Jordan M. Ruby**: Conceptualization; formal analysis; methodology; supervision; validation; writing – original draft; and writing – review & editing. **Alex Illescas**: Data curation; formal analysis; software; validation; visualization; writing – original draft; and writing – review & editing. **Haoyan Zhong**: Data curation; formal analysis; software; validation; visualization; writing – original draft; and writing – review & editing. **Kathryn R. DelPizzo**: Conceptualization; formal analysis; methodology; supervision; validation; and writing – review & editing. **Jashvant Poeran**: Formal analysis; methodology; supervision; validation; and writing – review & editing. **Jiabin Liu**: Formal analysis; methodology; supervision; validation; and writing – review & editing. **Crispiana Cozowicz**: Formal analysis; methodology; validation; and writing – review & editing. **Stavros G. Memtsoudis**: Conceptualization; formal analysis; methodology; project administration; supervision; validation; writing – original draft; and writing – review & editing. All authors have read and approved the final version of the manuscript. Stavros G. Memtsoudis had full access to all of the data in this study and takes complete responsibility for the integrity of the data and the accuracy of the data analysis.

## CONFLICTS OF INTEREST

S. G. M. is a one‐time consultant for Teikoku Pharma Inc. He has a US patent application for a Multicatheter Infusion System (US‐2017‐0361063) and is the owner of SGM Consulting, LLC, and co‐owner of Centauros Healthcare Analytics and Consulting, LLC. He is a partner in Parvizi Surgical Innovations, LLC, and an investor in HATH. None of the aforementioned relations influenced the conduct of the present study. None of the above relations influenced the conduct of the present project. The remaining authors declare no conflict of interest.

## ETHICS STATEMENT

This study is approved by the Institutional Review Board (IRB # 2016‐436).

## TRANSPARENCY STATEMENT

The lead author Stavros G. Memtsoudis affirms that this manuscript is an honest, accurate, and transparent account of the study being reported; that no important aspects of the study have been omitted; and that any discrepancies from the study as planned (and, if relevant, registered) have been explained.

## Data Availability

The authors confirm that the data supporting the findings of this study are available within the article.

## APPENDIX A

**Table A1:** Procedure codes for surgical interventions

Procedures	CPT code	ICD‐10 code
Shoulder and clavicle	23545, 23540, 23505, 23500, 23625, 23620, 23605, 23600, 23575, 23570, 23665, 23655, 23650, 23675, 23525, 23520, 23550, 23552, 23660, 23515, 23630, 23615, 23616, 23585, 23670, 23680, 23530, 23532	0PS5XX, 0PS6XX, 0PS7XX, 0PS8XX, 0PS9XX, 0PSBXX, 0RSGXX, 0RSHXX, 0RSJXX, 0RSKXX
Humerus and elbow	24577, 24576, 24565, 24560, 24505, 24500, 24620, 24655, 24650, 24640, 24535, 24530, 24675, 24670, 24605, 24600, 24615, 24579, 24575, 24515, 24546, 24545, 24635, 24586, 24587, 24665, 24666, 24685, 24582, 24566, 24538, 24516	0PSCXX, 0PSDXX, 0PSFXX, 0PSGXX, 0RSLXX, 0RSMXX
Forearm and wrist	25635, 25630, 25624, 25622, 25605, 25600, 25675, 25690, 25565, 25560, 25505, 25500, 25520, 25660, 25680, 25535, 25530, 25650, 25645, 25628, 25607, 25608, 25609, 25676, 25695, 25575, 25574, 25515, 25525, 25526, 25670, 25685, 25545, 25652, 25606, 25671, 25651	0PSHXX, 0PSJXX, 0PSKXX, 0PSLXX, 0PSMXX, 0PSNXX, 0RSNXX, 0RSPXX, 0RSQXX, 0RSRXX
Hand and finger	26742, 26740, 26675, 26670, 26641, 26645, 26755, 26750, 26775, 26770, 26605, 26600, 26607, 26705, 26700, 26725, 26720, 26746, 26686, 26685, 26665, 26765, 26785, 26615, 26715, 26735, 26676, 26650, 26756, 26776, 26608, 26706, 26727	0PSPXX, 0PSQXX, 0PSRXX, 0PSSXX, 0PSTXX, 0PSVXX, 0RSSXX, 0RSTXX, 0RSUXX, 0RSVXX, 0RSWXX, 0RSXXX
Pelvis and hip	27222, 27220, 27200, 27268, 27267, 27232, 27230, 27246, 27252, 27250, 27240, 27238, 27266, 27265, 27198, 27197, 27228, 27227, 27217, 27202, 27269, 27236, 27248, 27254, 27253, 27215, 27226, 27218, 27259, 27258, 27235, 27216, 27245, 27244, 27257, 27256	0QS2XX, 0QS3XX, 0QS4XX, 0QS5XX, 0QSSXX, 0SS5XX, 0SS6XX, 0SS7XX, 0SS8XX, 0SS9XX, 0SSBXX
Femur and knee	27517, 27516, 27510, 27508, 27502, 27500, 27538, 27552, 27550, 27562, 27560, 27520, 27503, 27501, 27532, 27530, 27519, 27514, 27507, 27506, 27513, 27511, 27540, 27557, 27558, 27556, 27566, 27524, 27509	0QS6XX, 0QS7XX, 0QS8XX, 0QS9XX, 0QSBXX, 0QSCXX, 0QSDXX, 0QSFXX, 0SSCXX, 0SSDXX
Leg and ankle	27842, 27840, 27810, 27808, 27788, 27786, 27825, 27824, 27762, 27760, 27768, 27767, 27781, 27780, 27831, 27830, 27752, 27750, 27818, 27816, 27848, 27846, 27814, 27792, 27829, 27828, 27826, 27827, 27766, 27769, 27784, 27832, 27758, 27823, 27822, 27756, 27759	0QSGXX, 0QSHXX, 0QSJXX, 0QSKXX, 0SSFXX, 0SSGXX
Foot and toe	28405, 28400, 28495, 28490, 28515, 28510, 28665, 28660, 28475, 28470, 28635, 28630, 28530, 28575, 28570, 28435, 28430, 28545, 28540, 28605, 28600, 28446, 28415, 28420, 28505, 28525, 28675, 28485, 28645, 28531, 28585, 28445, 28555, 28465, 28615, 28406, 28496, 28666, 28476, 28636, 28576, 28436, 28546, 28456, 29606, 28455	0QSLXX, 0QSMXX, 0QSNXX, 0QSPXX, 0QSQXX, 0QSRXX, 0SSHXX, 0SSJXX, 0SSKXX, 0SSLXX, 0SSMXX, 0SSNXX, 0SSPXX, 0SSQXX
ACL/UCL	24346, 24345, 29888	

Abbreviations: ACL, Anterior cruciate ligament; CPT, Current Procedural Terminology; ICD‐10, International Classification of Diseases, 10th Revision; UCL, Ulnar collateral ligament.
